# Akathisia induced by concurrent use of mirogabalin and sulfamethoxazole-trimethoprim: a case report

**DOI:** 10.1186/s40780-026-00578-y

**Published:** 2026-04-24

**Authors:** Takashi Nakashima, Kimitaka Suetsugu, Mina Nitta, Taichi Nagano, Makoto Yoshimitsu, Kenji Ishitsuka, Hideyuki Terazono

**Affiliations:** 1 Department of Clinical Pharmacy and Pharmacology, Graduate School of Medical and Dental Sciences, Kagoshima University, 8-35-1, Sakuragaoka, Kagoshima Japan; 2 Department of Pharmacy, Tarumizu Chuo Hospital, 1-140, Kinkocho, Tarumizu, Kagoshima Japan; 3 Department of Pharmacy, Kagoshima University Hospital, 8-35-1, Sakuragaoka, Kagoshima 890-8520 Japan; 4 Department of Hematology and Rheumatology, Graduate School of Medical and Dental Sciences, Kagoshima University, 8-35-1, Sakuragaoka, Kagoshima Japan

**Keywords:** Mirogabalin, Trimethoprim-sulfamethoxazole, Akathisia, Drug-drug interaction

## Abstract

**Background:**

Mirogabalin and trimethoprim-sulfamethoxazole (TMP/SMX) are frequently used as supportive purpose during and/or after chemotherapy. Mirogabalin is used to treat neuropathic pain, whereas TMP/SMX is used to prevent pneumocystis pneumonia (PCP) in patients who are immunocompromised. The interaction between mirogabalin and TMP/SMX has not been reported. Here, we report a case of akathisia that developed after concurrent administration of mirogabalin and TMP/SMX.

**Case presentation:**

We report the case of a 69-year-old male patient who experienced akathisia twice. The patient was diagnosed with diffuse large B-cell lymphoma and treated with the polatuzumab vedotin combined with rituximab, cyclophosphamide, doxorubicin, and prednisolone (Pola-R-CHP) regimen. On the fifth day of treatment, TMP/SMX at a dosage of 80 mg/400 mg once daily was initiated to prevent PCP. On day 20 of treatment, mirogabalin treatment was initiated for neuropathic pain probably due to polatuzumab vedotin. Akathisia first manifested on the day following the initiation of mirogabalin treatment, occurring 30 min after the administration of TMP/SMX. The patient experienced pruritus and dyspnea. Discontinuation of TMP/SMX resulted in the amelioration of symptoms. Akathisia occurred a second time, when TMP/SMX was resumed, resulting in itching of the upper body, symptoms of restlessness, and an inability to remain still. Based on these symptoms, the patient was diagnosed with akathisia. Following discontinuation of both TMP/SMX and mirogabalin, the symptoms improved. These episodes suggested the possibility that the concurrent use of mirogabalin and TMP/SMX caused akathisia. Blood concentration measurements obtained at symptom onset did not reveal a significant increase in mirogabalin levels, suggesting that the adverse event was unlikely to be explained solely by an increase in mirogabalin concentration.

**Conclusions:**

Concurrent use of mirogabalin and TMP/SMX may have been associated with the development of akathisia in the present case. Based on the potential for similar interactions, caution should be exercised when administering this drug to patients undergoing chemotherapy. To the best of our knowledge, this is the first case report describing akathisia associated with the concomitant use of mirogabalin and TMP/SMX, highlighting the need for careful monitoring of patients receiving both drugs.

**Supplementary Information:**

The online version contains supplementary material available at 10.1186/s40780-026-00578-y.

## Background

Mirogabalin exerts analgesic effects by binding to the α2δ subunit of voltage-dependent calcium channels in the nervous system, thereby inhibiting calcium currents. This inhibition activates the descending noradrenergic pain inhibitory pathway, making mirogabalin effective for the treatment of neuropathic pain [1]. The detailed mechanisms underlying chemotherapy-induced peripheral neuropathy (CIPN) remain largely unclear, despite being a common adverse event, and effective treatments have not been established. CIPN incidence is thought to vary depending on the treatment regimen. Colorectal cancer, breast cancer, gynecological cancers, and multiple myeloma show a CIPN incidence of 68.1% [2]. CIPN can hinder the continuation of treatment, and its prevention and management may improve cancer treatment outcomes and quality of life. However, discontinuation of the causative agent remains the primary approach for managing this condition [3, 4]. In patients undergoing chemotherapy with oxaliplatin and taxanes who experienced moderate-to-severe CIPN, mirogabalin reduced numeric pain rating scores by 30.9% [5]. Furthermore, previous reports suggested that mirogabalin is more effective than pregabalin, a drug with similar effects [6].

Trimethoprim-sulfamethoxazole (TMP/SMX) exerts antibacterial activity by inhibiting folate synthesis at two distinct points: SMX inhibits the synthesis of dihydrofolic acid by acting as a competitive inhibitor of para-aminobenzoic acid, whereas TMP inhibits dihydrofolate reductase and prevents folic acid activation. This dual inhibition results in a synergistic antibacterial effect, making TMP/SMX effective for prevention and treatment of *Pneumocystis carinii* pneumonia (PCP).The dosage of TMP/SMX differs between prophylaxis and treatment: for prophylaxis, a standard dose of 80 mg/400 mg is typically taken once daily, while for treatment, doses range from 720 mg/3600 mg to 960 mg/4800 mg, divided into three administrations per day. A meta-analysis of randomized controlled trials involving patients with hematologic malignancies and organ transplant recipients demonstrated that prophylactic administration of TMP/SMX reduced the risk of developing PCP by 91% [7]. Furthermore, TMP/SMX is considered to have a higher prophylactic efficacy than inhaled pentamidine or oral atovaquone, which are also used as preventive treatments for PCP [8]. The risk of PCP is high in patients who are immunosuppressed. According to the guidelines for managing febrile neutropenia, prophylactic administration of TMP/SMX is recommended in patients undergoing rituximab combination therapy to prevent PCP [9].

The drugs mirogabalin and TMP/SMX are commonly used in patients undergoing chemotherapy. We present a previously unreported case of akathisia that occurred when both drugs were administered simultaneously.

## Case presentation

The patient was a 69-year-old male with a medical history of hypertension. The patient has a history of allergies, specifically a generalized skin rash without respiratory symptoms following administration of penicillin and cephalosporin antibiotics. The present ailments of the patient comprised pain in the left flank and contiguous back one month prior to visiting our hospital. A local physician identified a splenic tumor, and aspiration cytology of the splenic tumor led to a diagnosis of diffuse large B-cell lymphoma. Positron emission tomography showed the accumulation of FDG in the tumor of spleen and retroperitoneal area. The patient was referred to our hospital. A combination of polatuzumab vedotin combined with rituximab, cyclophosphamide, doxorubicin, and prednisolone (Pola-R-CHP) was initiated [10, 11] (Table 1). Upon admission, the patient’s liver function tests showed 24 IU/L aspartate aminotransferase, 26 IU/L alanine aminotransferase, and 0.5 mg/dL total bilirubin (T-Bil). Renal function tests indicated a creatinine level of 0.72 mg/dL and creatinine clearance rate of 86.28 mL/min. Liver and kidney functions were normal. The patient was on a regular medication regimen consisting of amlodipine 5 mg once daily after breakfast and magnesium oxide 330 mg three times daily after each meal. Additionally, loxoprofen sodium 60 mg and rebamipide 100 mg were prescribed to be taken as needed for pain, one tablet each per dose.

**Table 1:**
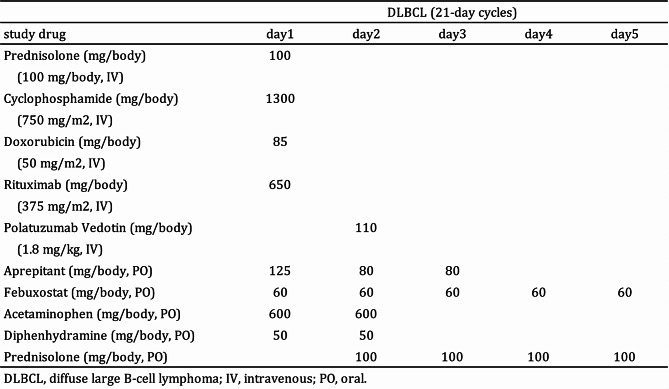
Study drug administration

**Fig. 1:**
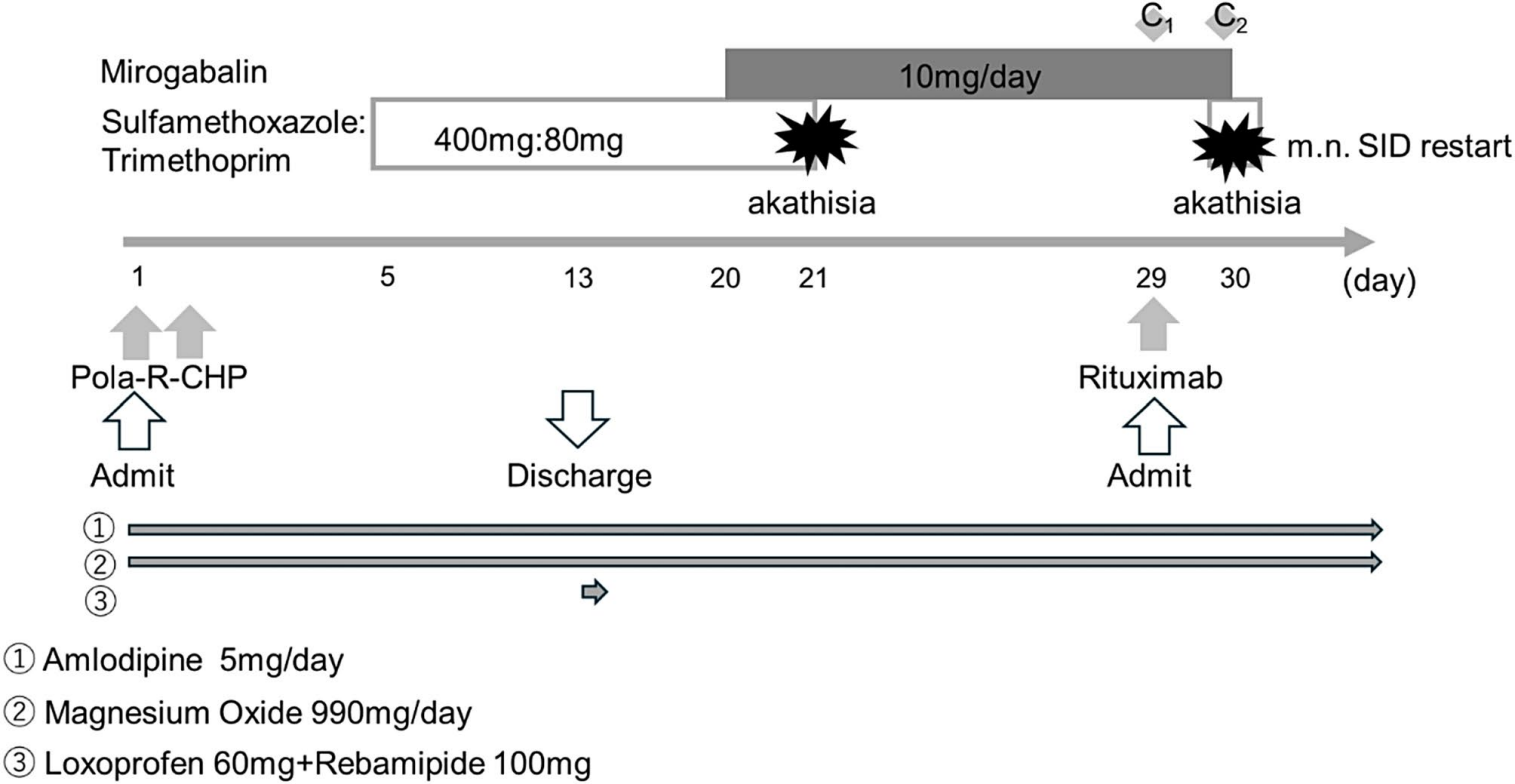
Timeline of treatment, drug administration, and akathisia onset. This figure illustrates the temporal relationship between chemotherapy administration (the Pola-R-CHP regimen and rituximab monotherapy), mirogabalin, and trimethoprim/sulfamethoxazole (TMP/SMX), in relation to the onset of akathisia. C1 and C2 indicate the first and second plasma concentration measurements of mirogabalin, respectively. Numbers ①–③ represent concomitant medications that were continuously administered throughout the hospitalization period: ① amlodipine 5 mg/day, ② magnesium oxide 990 mg/day, and ③ loxoprofen 60 mg and rebamipide 100 mg. The clinical course from admission to discharge, including drug discontinuation and re-administration, as well as the timing of akathisia onset, is presented along a day-based timeline. During the second cycle, polatuzumab vedotin was discontinued due to the development of peripheral neuropathy, and the treatment regimen was modified to R-CHP. Akathisia recurred after TMP/SMX was reintroduced on the day following rituximab administration, and consequently, prednisolone, cyclophosphamide, and doxorubicin were not administered during that cycle

The drug administration is shown in Fig. 1. On day 5 of the first Pola-R-CHP cycle, prophylaxis for PCP was initiated with 80 mg/400 mg TMP/SMX once daily after breakfast. No major adverse events were observed during inpatient treatment. The patient was discharged on day 13 of the first cycle with plans to continue treatment as an outpatient from the second cycle onward. On day 20 of the first cycle, the patient developed numbness and pain extending from the fingertips to the shoulder. These symptoms interfered with activities of daily living, for example, wearing a belt, and caused sleep disturbance due to their severity, prompting an outpatient visit. Upon clinical evaluation, the condition was diagnosed as polatuzumab vedotin–induced peripheral sensory neuropathy and was graded as Grade 3 according to the Common Terminology Criteria for Adverse Events (CTCAE). Consequently, polatuzumab vedotin was discontinued from the second cycle onward. Mirogabalin was prescribed at a dose of 5 mg twice daily after breakfast and dinner. The medication was taken by the patient in the afternoon and at midnight on the same day. On the morning of day 21 of the first cycle, the patient reported pruritus and a sensation of being unable to breathe after receiving TMP/SMX and subsequently visited a nearby clinic. The symptoms were improved by administration of antihistamines and steroids. Subsequently, the patient decided to discontinue use of TMP/SMX by himself, while continuing to take mirogabalin. The start of the second cycle was postponed.

On day 1 of the second cycle (day 29 of the first cycle), rituximab was administered in an outpatient setting and TMP/SMX was resumed. On day 2 of the second cycle (day 30 of the first cycle), 30 min after taking TMP/SMX, the patient reported experiencing pruritus in the upper body, a constricting sensation, and the feeling of insects crawling all over his body. Feelings of restlessness and an inability to remain stationary were also noted and these symptoms were declared to be similar to those observed on day 21 of the first cycle by the patient. On the same day, the patient was admitted for observation, and chemotherapy; mirogabalin and TMP/SMX were discontinued. Chlorpheniramine injection (5 mg), a combination of glycyrrhizin, glycine, and cysteine (20 mL), and prednisolone injection (20 mg) were administered; however, the symptoms did not improve. The patient also reported generalized pain, and an acetaminophen injection (500 mg) was administered. Additionally, hydroxyzine (25 mg) was administered at night and again in the early morning of the following day. On day 3 of the second cycle (day 31 of the first cycle), all symptoms resolved. No similar symptoms were observed after discontinuation of TMP/SMX and mirogabalin. The neurologist diagnosed the patient with the onset of akathisia. Because akathisia recurred shortly after the concomitant administration of mirogabalin and TMP/SMX, the physician suspected that an increase in mirogabalin concentration might have contributed to the adverse event. Therefore, plasma concentrations of mirogabalin were measured using residual samples obtained during routine clinical blood sampling after the onset of akathisia. Blood concentrations of mirogabalin were measured twice. The first measurement was taken 2 h after administration of mirogabalin on day 1 of the second cycle (day 29 of the first cycle), revealing a concentration of 7.71 ng/mL (C1). A second measurement was taken 5 h after mirogabalin administration on day 2 of the second cycle (day 30 of the first cycle), showing a concentration of 29.7 ng/mL (C2).

Regarding the subsequent clinical course, on day 29 (Day 1 of the second chemotherapy cycle), rituximab monotherapy was administered; however, akathisia recurred on the following day. Consequently, prednisolone, cyclophosphamide, and doxorubicin were withheld for that cycle, and both mirogabalin and the TMP/SMX combination were discontinued. As a treatment strategy following the recurrence of akathisia, polatuzumab vedotin was discontinued, and R-CHP therapy without polatuzumab vedotin was continued thereafter. From day 51(Day 1 of the third cycle), this modified regimen was resumed as scheduled and was subsequently administered every 21 days, with a total of six cycles completed. With regard to CIPN, the risk was considered to be markedly reduced after discontinuation of polatuzumab vedotin; therefore, mirogabalin was not reintroduced from the second cycle onward. Regarding prophylaxis for PCP, re-initiation of the TMP/SMX combination was avoided due to concerns about the risk of recurrent akathisia. The use of alternative prophylactic agents was considered but ultimately not pursued at the discretion of the treating physician. As a result, no episodes of PCP occurred, and no recurrence of akathisia was observed during cycles 3 through 6.

## Discussion and conclusions

This case represents the first reported instance of akathisia that occurred reproducibly with concurrent use of mirogabalin and TMP/SMX.

Akathisia is characterized by motor hyperactivity, intense anxiety, agitation, and internal restlessness. The underlying mechanism is thought to differ from that of other extrapyramidal symptoms associated with the substantia nigra-striatal system, one of which is the antagonistic effect of dopamine on the mesolimbic and mesocortical pathways [12]. The typical subjective symptom of akathisia is a strong, compelling urge to move the limbs or entire body. Patients often have difficulty keeping their feet still, and when forced to remain stationary, their internal restlessness intensifies. Although akathisia itself is not directly life-threatening, if left untreated, the associated anxiety and agitation may worsen, potentially leading to self-harm or suicidal behavior [13].

Because the patient had been taking TMP/SMX during hospitalization, it was not suspected as a cause of the current symptoms. Allergic reactions to food were also assessed. After symptom onset during the first occurrence (day 20 of the first cycle), mirogabalin treatment was continued without significant issues until the second occurrence of symptoms (days 2 and 30 of the second and first cycles, respectively). During the first symptom of this episode, akathisia manifested 30 min after administration of TMP/SMX, which was administered 8 h after the mirogabalin dose. In the second episode, akathisia symptoms appeared 30 min after TMP/SMX administered 30 min after mirogabalin administration.

Mirogabalin is excreted renally and its renal clearance rate is higher than its glomerular filtration rate. Mirogabalin is secreted and excreted from the renal tubules, and its plasma concentrations increase in patients with impaired renal function [14]. In healthy individuals taking mirogabalin at a dose of 5 mg twice daily, the maximum plasma concentration (Cmax) was reported to be 97 ng/mL, with a time to reach this concentration of 1 h. In contrast, a single 50 mg dose, associated with a significantly higher incidence of adverse events, resulted in a Cmax of 671 ng/mL. Adverse events such as drowsiness and dizziness have been reported to occur in a concentration-dependent manner [15]. Reports on plasma concentration parameters in healthy Asian individuals are generally consistent, indicating that the Cmax following a single dose of 5 mg was 89.8 ng/mL. Even at a higher dose of 15 mg administered twice daily, the Cmax was 290 ng/mL, with no treatment required for adverse events [16]. No renal impairment was observed in the present case, and the plasma concentration of mirogabalin was lower than that associated with a higher incidence of adverse events. These findings suggest that the adverse event was unlikely to be explained solely by an increase in the mirogabalin concentration.

There are no reports of akathisia associated with mirogabalin; however, it has been documented that gabapentinoids may cause extrapyramidal disorders. In addition, duloxetine carries a risk of extrapyramidal disorders [17]. The mechanism of action suggests that duloxetine impairs dopamine function and has the potential to cause extrapyramidal disorders via activation of norepinephrine. Additionally, extracellular release of the excitatory amino acid glutamate may contribute to the inhibition of neuronal excitability [18]. Furthermore, pregabalin, which shares a similar mechanism of action with mirogabalin, has been reported to cause akathisia. Akathisia was reported to develop after a single dose of pregabalin, which improved after switching to gabapentin [19]. This result suggests that functional alterations in neurotransmitter systems, including GABAergic pathways, associated with pregabalin administration may contribute to the development of akathisia [20].

Although psychiatric symptoms associated with prednisolone (PSL) have been reported [21], no previous reports of akathisia have been identified in association with Pola-R-CHP therapy, its concomitant agents, or the regular and supportive medications used in this case. In the present case, the initial onset of akathisia was observed on day 20 following the administration of Pola-R-CHP therapy, while the second episode occurred on day 30, the day after administration of rituximab monotherapy. At that time, no agents other than rituximab included in the Pola-R-CHP regimen were administered. Furthermore, subsequent cycles were administered as R-CHP therapy with discontinuation of polatuzumab, after which no recurrence of akathisia was observed. Taken together, these clinical observations—namely, (1) the 20-day interval between Pola-R-CHP administration and the initial onset of akathisia, (2) the occurrence of recurrence during rituximab monotherapy alone, and (3) the absence of recurrence following discontinuation of polatuzumab during subsequent R-CHP therapy—suggest that Pola-R-CHP therapy itself was unlikely to be directly involved in the development of akathisia. In addition, the patient’s regular medications, including amlodipine and magnesium oxide, were continued during both TMP/SMX coadministration and mirogabalin coadministration; however, no episodes of akathisia were observed under these conditions. Based on these findings, the concomitant use of mirogabalin and TMP/SMX was considered the most plausible suspected drug–drug interaction contributing to the development of akathisia in this case.

In this study, the relationship between concurrent use of mirogabalin and TMP/SMX and the occurrence of akathisia was objectively assessed using the Naranjo algorithm. The Naranjo scale was developed to standardize the evaluation of causal relationships for all drug-related adverse reactions and is widely used both domestically and internationally [22]. The Naranjo Adverse Drug Reaction Probability Scale consists of ten questions that can be answered with “yes,” “no,” or “unknown.” Each response was assigned a different value (-1, 0, + 1, or + 2) (Additional file 1). The total score ranges from − 4 to + 13. A score of 9 or higher indicates a definite reaction, a score between 5 and 8 suggested a high probability of a reaction, a score between 1 and 4 indicates a possible reaction, and a score of 0 or lower indicates a doubtful reaction. In the present case, investigation of the causal relationship between the medication and adverse events using the Naranjo algorithm yielded a score of 7, suggesting a probable association between akathisia adverse events and the interaction resulting from concurrent use of mirogabalin and TMP/SMX.

Although the mechanisms underlying akathisia are not fully understood, reports have indicated that TMP/SMX can penetrate the central nervous system [23]. Additionally, for every 1 mg/kg increase in the daily dose of TMP, the risk of developing acute psychosis increases by 40% [24]. In this case, the plasma concentration of TMP could not be measured; therefore, the possibility that the onset of akathisia is associated with an increase in TMP concentration cannot be ruled out. However, given that the patient was taking TMP/SMX at a prophylactic dose, it is more likely that other factors contributed rather than an elevation in plasma concentration. Furthermore, TMP/SMX may disrupt the gut microbiota, that produces D-alanine, an essential component of N-methyl-d-aspartate receptors, thereby reducing the activity of these receptors [25]. Additionally, inhibition of folate production in the central nervous system has been implicated as a trigger for psychiatric symptoms [26]. In this case, mirogabalin and TMP/SMX may have acted concurrently within the central nervous system through distinct pharmacological mechanisms. These mechanisms may theoretically exert additive or synergistic influences on dopaminergic pathways and thereby potentially contribute to the development of akathisia; however, this hypothesis remains speculative and requires further investigation.

The findings derived from this case are subject to several limitations in interpretation. This was a retrospective case report, and planned blood concentration measurements before and after symptom onset could not be performed. In particular, plasma concentrations of TMP/SMX were not measured, and comprehensive pharmacokinetic evaluation of mirogabalin, including trough and peak levels, was not conducted. Therefore, based on the present data alone, it is difficult to clearly distinguish the relative contribution of pharmacokinetic interactions, including concentration changes during concomitant use, from pharmacodynamic effects. In light of these limitations, the interaction mechanism and causal relationship cannot be definitively established from this case alone, and further accumulation of similar cases and prospective measurements are warranted to elucidate the mechanism underlying akathisia. Nevertheless, considering that akathisia occurred exclusively during concomitant administration of mirogabalin and TMP/SMX, recurred reproducibly upon re-challenge, and was supported by the Naranjo Scale assessment (score of 7, indicating a probable association), this case provides clinically meaningful information suggesting a possible association with the concomitant use of these two agents.

In conclusion, although interactions between mirogabalin and TMP/SMX have not been extensively reported, the concomitant use of these medications may have contributed to the development of akathisia in the present case. Peripheral neuropathy is a common side effect during chemotherapy, necessitating careful management because of its association with a decline in activities of daily living. Measures are needed to prevent PCP in patients undergoing some regimens of chemotherapy, particularly those with a compromised immune system. In patients undergoing chemotherapy, mirogabalin and TMP/SMX may be administered concurrently. Therefore, careful attention is required regarding the potential development of adverse events. To our knowledge, this is the first case report of akathisia associated with the concomitant use of mirogabalin and TMP/SMX, and it underscores the importance of awareness of akathisia in patients receiving both medications. This case should be interpreted as a hypothesis-generating observation, and further accumulation of similar cases will be necessary to clarify the potential interaction between these medications.

## Electronic Supplementary Material

Below is the link to the electronic supplementary material.

Supplementary Material 1

## Data Availability

Not applicable.

## Abbreviations

TMP/SMX: Trimethoprim-sulfamethoxazole
PCP: Pneumocystis pneumonia
Pola-R-CHP: Polatuzumab vedotin combined with rituximab, cyclophosphamide, doxorubicin, and prednisolone
