# Supplementary material for: Akathisia induced by concurrent use of mirogabalin and sulfamethoxazole-trimethoprim: a case report
Source: J Pharm Health Care Sci. 2026 Apr 24;12:47. doi: 10.1186/s40780-026-00578-y (PMC13134217; doi:10.1186/s40780-026-00578-y)
Supplement: Supplementary file 1 — Supplementary Material 1 [file 40780_2026_578_MOESM1_ESM.docx]

**Additional file 1.**

**File format including the correct file extension for example .pdf, .xls, .txt, .pptx (including name and a URL of an appropriate viewer if format is unusual)**

**Title of data: Naranjo Adverse Drug Reaction Probability Scale Score for this case.**
